# A cost-effectiveness analysis of Molnupiravir and Paxlovid for outpatient treatment of COVID-19 in three African countries

**DOI:** 10.4102/jphia.v16i1.805

**Published:** 2025-06-09

**Authors:** Ijeoma P. Edoka, Tom Drake, Peter Baker, Raji Tajudeen, Elias Asfaw, Javier Guzman, Nicaise Ndembi, Justice Nonvignon, Jean Kaseya

**Affiliations:** 1 Health Economics and Epidemiology Research Office (HE2RO), Department of Internal Medicine, Faculty of Health Sciences, University of the Witwatersrand, Johannesburg, South Africa; 2 School of Public Health, Faculty of Health Sciences, University of the Witwatersrand, Johannesburg, South Africa; 3 Center for Global Development, London, United Kingdom; 4 Africa Centres for Disease Control and Prevention (Africa CDC), Addis Ababa, Ethiopia; 5 School of Public Health, University of Ghana, Legon, Ghana

**Keywords:** cost-effectiveness analysis, COVID-19 oral antivirals, resource allocation, COVID-19, healthcare decision making

## Abstract

**Background:**

Two COVID-19 oral antivirals (COAVs), Molnupiravir and Paxlovid, have been shown to be cost-effective in high-income countries.

**Aim:**

This study assesses the cost-effectiveness of Paxlovid and Molnupiravir, compared to usual care in three African countries.

**Setting:**

The study was conducted using data from Ghana, Rwanda and Zambia.

**Methods:**

We modelled costs (2022 United States dollars) and health outcomes in the acute phase of COVID-19 from a public payer’s perspective in three unvaccinated target populations: (1) all adult patients, (2) patients aged 65 years and above (elderly), and (3) adult patients with other underlying risk factors for disease severity. We conducted pairwise and full incremental analyses.

**Results:**

In the pairwise analysis, Paxlovid was less costly and more effective than usual care (i.e. dominated) in all three study countries for elderly patients, while in adults with other underlying risk factors, Paxlovid dominated in Rwanda and Zambia, and Molnupiravir dominated usual care in Rwanda. Neither Paxlovid nor Molnupiravir were cost-effective in the all-adult group in any country context. In the full incremental analysis, Paxlovid dominated both Molnupiravir and usual care in elderly patients (in all three countries) and in adults with other risk factors (in Rwanda and Zambia). Key determinants of cost-effectiveness were COAV price, likelihood of early treatment initiation, hospitalisation rates and vaccination status.

**Conclusion:**

In African settings like Zambia, Ghana or Rwanda, Paxlovid could be cost-effective in unvaccinated populations and those at high risk of progression to severe COVID-19.

**Contribution:**

This study broadly supports African governments decisions not to procure substantial quantities of COAV.

## Introduction

COVID-19 had a profound impact across many African countries, not only through its direct effect on lives and livelihoods but also indirectly by straining health systems and diverting resources from other essential health services.^1,2^

The global roll-out of COVID-19 vaccines contributed significantly to reducing the health and economic impact of COVID-19. In addition, therapeutic interventions for the clinical management of COVID-19, both in the outpatient and inpatient settings, have become available,^3^ offering more options in the fight against the disease. Two COVID-19 oral antivirals (COAVs), Pfizer’s nirmatrelvir and ritonavir (Paxlovid) and Merck’s Molnupiravir have been recommended for use in several countries including African Union member states.^4^ Initiatives such as the voluntary licensing agreement between Pfizer, Merck and the Medicines Patent Pool and the test and treat programmes announced by the United States Agency for International Development (USAID) and the COVID-19 Treatment Quick Start Consortium in 2022 have made available low-priced, generic versions of patent-protected medicines and donations for low- and middle-income countries (LMICs) use. When compared to placebos, these COAVs have been shown to reduce the risk of hospitalisations and deaths in unvaccinated outpatients with at least one risk factor for progression to more severe conditions.^5,6^ Given the low levels of vaccination coverage in Africa, the availability and use of these therapeutic agents could be important for minimising the impact of COVID-19 on individuals and fragile health systems in Africa. Initiatives such as the Accord initiative by Pfizer and the voluntary licensing agreement between Pfizer, Merck and the Medicines Patent Pool could see the manufacture of affordable generic versions of patent-protected medicines on a not-for-profit basis in several LMICs at prices significantly lower than is offered to high-income countries.^7,8^

Decision-makers in LMICs will need to weigh various factors, including clinical impact, cost, availability and feasibility of use, in deciding to recommend COAVs for use within their context. Given growing budget constraints facing many LMICs, not least because of the COVID-19 pandemic, an understanding of the value for money of COAVs is likely to be crucial to informing policy decisions on the use of COAVs in these settings. Some evidence suggests that Paxlovid and Molnupiravir can represent good value for money in some contexts.^9,10,11^ However, these studies largely focus on populations in high-income countries and, given differences between settings along dimensions that can influence results of cost-effectiveness analyses, these findings may not be transferable to LMICs. For example, the burden of disease (hospitalisations and deaths) in Africa has been markedly lower compared to other regions and could result in less favourable cost-effectiveness outcomes. On the other hand, the higher probability of death following hospitalisation in Africa because of poor health systems^12^ may mean that the value of COAVs, in terms of reducing the need for hospitalisation, may be greater in the African setting. To be effective, COAV treatment must be initiated within 5 days of symptom onset.^5,6,13^ Therefore, the lower likelihood of early treatment initiation may limit the effectiveness of COAVs in LMICs where COVID-19 testing rates and testing policies may not be as rigorous as in high-income countries to allow early diagnosis and treatment initiation. Cost-effectiveness analyses that consider the nuances within the African context are crucial for understanding the conditions under which COAVs would represent good value for money for African countries.

## Research methods and design

This study assesses the cost-effectiveness of Paxlovid and Molnupiravir in unvaccinated adult populations, including sub-populations at high risk of disease progression in three African countries. The study countries were selected to reflect regional distribution of countries within the African Union and represent some factors that may directly or indirectly impact the effectiveness and thus, the cost-effectiveness of COAVs. These include the probability of early treatment initiation and COVID-19 vaccination coverage. Countries were stratified by vaccination coverage and COVID-19 test rates (a proxy for early diagnosis and treatment)^14^ and three countries – Ghana, Rwanda and Zambia – were selected from each stratum (Online Appendix 1 Figure S1, Table S1). However, it should be noted that cost-effectiveness results obtained from these three counties may not necessarily be generalisable to other countries within each stratum because of other context-specific parameters that are likely to vary between countries. Below, we describe these parameters, and a series of sensitivity analyses we conducted to assess the impact of uncertainties in these parameters on our findings. This would aid decision-makers in adapting findings from this study to their settings.

The cost-effectiveness analysis was conducted from a public healthcare payer’s perspective. Given heterogeneity of the trial design, baseline characteristics of trial participants and predominant COVID-19 variant,^5,6,13^ we conducted a pairwise analysis as our base case analysis by comparing the costs and health outcomes of a 5-day course of Paxlovid (Nirmatrelvir 300 mg + Ritonavir 100 mg twice daily) to usual care (when no COAV treatment is administered) and a 5-day course of Molnupiravir (800 mg twice daily) to usual care. However, we also conducted a full, incremental analysis involving a head-to-head comparison of Paxlovid, Molnupiravir and usual care. A decision tree was constructed in Microsoft^®^ Excel to model adult patients with mild or moderate symptoms of COVID-19 who were treated in the outpatient setting. The model estimated health outcomes and costs in three target populations: (1) all unvaccinated adult patients, (2) unvaccinated patients aged 65 years and above, and (3) unvaccinated adult patients with at least one other underlying risk factors for COVID-19 disease severity.

We modelled disease progression in the acute phase of the disease (30 days - study time horizon). Patients enter the model either remaining in the outpatient setting or progressing to hospitalisation (Online Appendix 1 Figure S2). Those remaining in the outpatient setting either survive or die from other causes. For those hospitalised, we simulated different levels of care depending on disease severity – mild or moderate, severe and critical – and their corresponding survival rates. Given the need for treatment initiation within 5 days of symptom onset, we explicitly modelled the probability of early initiation using country-specific COVID-19 test rates as proxies.^15^ We assumed no treatment effect if treatment was initiated after 5 days of symptom onset.^5,6,13^

In this study, we estimated both incremental cost-effectiveness ratios (ICERs) and incremental net monetary benefits (INMBs). The ICER compared the difference between costs and health outcomes of the COAV and comparator. It was estimated as a ratio of the difference in costs and the difference in health outcomes and expressed as additional or incremental cost per health gain.^16^ For the INMB, health gains or incremental health outcomes were rescaled to monetary terms by multiplying the difference in health outcomes with a cost-effectiveness threshold.^16^ We estimated INMB as the difference in rescaled health gain and incremental cost.^16^ A COAV was regarded as cost-effective when INMB was positive at the assumed cost-effectiveness threshold. In this study, we used country-specific cost-effectiveness thresholds that reflect the estimated marginal productivity of each study country’s health system^17^ (Table 1). These thresholds were chosen because they represent the health opportunity cost of resource allocation decisions more appropriately.

**TABLE 1:** Model parameters (base case).

Parameters	Ghana		Rwanda		Zambia		Distribution	Data source
	Base case	s.e.	Base case	s.e.	Base case	s.e.		
**Disease parameters**								
Care initiated within 5 days of symptom onset (%)	7	1	41	8	18	4	Beta	Hasell et al.^15^
**Hospitalisation rates (%)**								
All adult population	3	1	1	0.3	2	0.4	Beta	Ayisi-Boateng et al.^18^, Ministry of Health Zambia^19^, Jassat et al.^21^, World Health Organization African Region^22^
Adults ≥ 65 years old	25	5	12	2	18	4	Beta	Ayisi-Boateng et al.^18^, Ministry of Health Zambia^19^, Jassat et al.^21^, World Health Organization African Region^22^
Adults other risk factors	8	2	4	1	6	1	Beta	Ayisi-Boateng et al.^18^, Ministry of Health Zambia^19^, Jassat et al.^21^, World Health Organization African Region^22^
**Disease severity (%)**								
Hospitalised, mild or moderate	7	14	53	11	62	12	Dirichlet	Ayisi-Boateng et al.^18^, Ministry of Health Zambia^19^, Rwanda Biomedical Centre^20^
Hospitalised, severe	23	5	10	2	25	5	Dirichlet	Ayisi-Boateng et al.^18^, Ministry of Health Zambia^19^, Rwanda Biomedical Centre^20^
Hospitalised, critical	7	1	37	7	13	3	Dirichlet	Ayisi-Boateng et al.^18^, Ministry of Health Zambia^19^, Rwanda Biomedical Centre^20^
**Mortality rate (%)**								
Outpatient	4	1	3	1	5	1	Beta	World Health Organization^30^
Hospitalised, mild or moderate	4	1	3	1	5	1	Beta	World Health Organization^30^
Hospitalised, severe	17	3	21	4	19	4	Beta	Biccard et al.^12^, Ayisi-Boateng et al.^18^, Rwanda Biomedical Centre^20^
Hospitalised, critical	26	5	21	4	34	7	Beta	Biccard et al.^12^, Ayisi-Boateng et al.^18^, Rwanda Biomedical Centre^20^
**Length of hospital stay (days)**								
Outpatient care only	5	1	5	1	5	1	Gamma	Jassat et al.^21^, Crankson et al.^23^
Mild or moderate, survived	3	0	3	0	3	0	Gamma	Jassat et al.^21^, Crankson et al.^23^
Mild or moderate, died	5	0	5	0	5	0	Gamma	Jassat et al.^21^, Crankson et al.^23^
Severe, survived	5	0	5	0	5	0	Gamma	Jassat et al.^21^, Crankson et al.^23^
Severe, died	3	0	3	0	3	0	Gamma	Jassat et al.^21^, Crankson et al.^23^
Critical, survived	5	0	5	0	5	0	Gamma	Jassat et al.^21^, Crankson et al.^23^
Critical, died	3	0	3	0	3	0	Gamma	Jassat et al.^21^, Crankson et al.^23^
**Clinical efficacy**								
**Clinical efficacy (Paxlovid)**								
Relative risk, hospitalisation	0.15	0.054	0.15	0.054	0.15	0.054	Log-normal	Hammond et al.^5^
Relative risk, mortality in hospitalised patients	0.12	0.082	0.12	0.082	0.12	0.082	Log-normal	Hammond et al.^5^
**Clinical efficacy (Molnupiravir)**								
Relative risk, hospitalisation	0.67	0.11	0.67	0.11	0.67	0.11	Log-normal	Jayk Bernal et al.^6^
**Costs**								
**Cost of Illness (2022 $), per patient/day**								
Outpatient care only	20.93	4.19	12.49	2.50	17.59	3.52	Gamma	Ismaila et al.^28^, Torres-Rueda et al.^29^
Mild or moderate	581.19	116.24	502.41	100.48	583.45	116.69	Gamma	Ismaila et al.^28^, Torres-Rueda et al.^29^
Severe	1363.70	272.74	1178.85	235.77	1369.00	273.80	Gamma	Ismaila et al.^28^, Torres-Rueda et al.^29^
Critical	1570.36	314.07	1517.06	303.41	1590.55	318.11	Gamma	Ismaila et al.^28^, Torres-Rueda et al.^29^
**Treatment costs (full course, $)**								
Paxlovid	25	-	25	-	25	-	-	Ledford^8^
Molnupiravir	20	-	20	-	20	-	-	Hill et al.^7^
**Health outcome parameters**								
**Average target population age (years)**								
All adult population	49	-	49	-	49	-	-	Assumption
Adults ≥ 65	65	-	65	-	65	-	-	Assumption
Adults with comorbidities	49	-	49	-	49	-	-	Assumption
**Remaining life expectancy (years)**								
All adult population	28		30	-	27	-	-	World Health Organization^30^
Adults ≥ 65	14	-	15	-	14	-	-	World Health Organization^30^
Adults with comorbidities	28	-	30	-	27	-	-	World Health Organization^30^
**Disability weight**								
Outpatient care only	0.051	0.011	0.051	0.011	0.051	0.011	Beta	Wyper et al.^31^
Mild or moderate	0.133	0.026	0.133	0.026	0.133	0.026	Beta	Wyper et al.^31^
Severe	0.655	0.038	0.655	0.038	0.655	0.038	Beta	Wyper et al.^31^
Critical	0.655	0.038	0.655	0.038	0.655	0.038	Beta	Wyper et al.^31^
**Cost-effectiveness threshold ($)**								
Marginal health system productivity	433.25	-	246.50	-	503.50	-	-	Ochalek et al.^17^

Note: Please see the full reference list of the article Edoka IP, Drake T, Baker P, et al., A cost-effectiveness analysis of Molnupiravir and Paxlovid for outpatient treatment of COVID-19 in three African countries. J Public Health Africa. 2025;16(1), a805. https://doi.org/10.4102/jphia.v16i1.805, for more information.

s.e. standard error, $, United States dollar.

### Model parameters

The model was populated using parameters obtained from various sources (Table 1).

#### Disease parameters

For the adult population, country-specific disease parameters, including hospitalisation rates, in-hospital disease severity and mortality rates, were obtained from publicly available sources or literature that reported on the disease’s epidemiological profile during the pandemic’s first phases.^12,18,19,20,21,22,23^ Considering the epidemiologic profile of the disease in Africa at the time of the study, that is, potentially high natural immunity and the dominant milder COVID-19 Omicron variant,^21,24,25^ we adjusted hospitalisation rates, inpatient disease severity and length of hospitalisation downwards based on the relative disease severity between earlier COVID-19 variants and the Omicron variant.^21^ For older patients and patients with underlying risk factors, hospitalisation rates were assumed to be 9 and 3 times higher than hospitalisation rates in the overall adult population, respectively.^26^

#### Clinical efficacy

Clinical efficacy of Paxlovid and Molnupiravir was modelled as a reduction in hospitalisation risk following COAV treatment initiation within 5 days of symptom onset in unvaccinated patients.^5,6^ In addition, we modelled a reduction in the risk of in-hospital mortality for Paxlovid but not for Molnupiravir given the limited evidence on the efficacy of Molnupiravir on in-hospital mortality.^6^ Clinical efficacy of each COAV was obtained from a meta-analysis of existing randomised control trials.^27^

#### Costs

Costs of clinical management of COVID-19 were obtained from existing literature. For Ghana, cost estimates were obtained from a primary costing study reporting context-specific estimates based on national treatment guidelines for outpatient and inpatient management of COVID-19, disaggregated by disease severity.^28^ In the absence of context-specific cost estimates for Rwanda and Zambia, clinical management costs were obtained from Torres-Rueda et al.^29^ Torres-Rueda et al.^29^ estimated COVID-19 clinical management costs for 79 LMICs by extrapolating unit costs from three LMICs. However, a comparison to Ghana-specific cost estimates^28^ suggests that unit costs from Torres et al. may have been underestimated. Both Ismaila et al.^28^ and Torres-Rueda et al.^29^ included diagnostics, medication, staff time and overhead costs in estimating COVID-19 clinical management cost. However, whilst Torres-Rueda adopted a modelling approach to extrapolate costs to multiple countries, Ismaila et al. adopted a normative approach using country-specific data. Therefore, clinical management costs for Rwanda and Zambia were scaled up using the proportionate difference between Torres-Rueda et al.^29^ cost estimates for Ghana and the reported context-specific cost for Ghana.^28^ Given the uncertainty in the estimates of clinical management costs, we assessed this in sensitivity analyses.

A full 5-day course of Paxlovid was assumed to cost $25.00 based on a maximum price per course negotiated by Clinton Health Access Initiative for the generic version of Paxlovid.^8^ A full course of Molnupiravir was assumed to costs $20.00 based on pricing discounts offered to LMICs through a voluntary licensing agreement between Merck and the Medicines Patent Pool (MPP) to allow the manufacture of a cheaper generic version of Molnupiravir for LMICs.^7^ All costs were expressed in 2022 US$.

#### Health outcomes

Health outcomes were expressed as disability-adjusted life years (DALYs), which were estimated as the sum of years of life lost (YLL) and years lived with disability (YLD). Remaining life expectancy at time of death for each country was obtained from the World Health Organization (WHO) life tables.^30^ We used disability weights for each health state recommended by the European Burden of Disease Network Consensus COVID-19 model^31^ based on the Global Burden of Disease Study 2019 (GBD 2019)^32^ and the European Disability Weight Study^33^ descriptions of infectious diseases of the lower respiratory tract (LRT). We applied disability weight defined for moderate LRT disease requiring community care for our study outpatient care health state; disability weight defined for severe hospitalised non-intensive care for our study mild or moderate inpatient health state; and disability weight for critical hospitalised intensive care LRT disease for both our study severe and critical inpatient health states. Given that we modelled the acute phase of the COVID-19 disease (30-day time horizon), YLD was not discounted. However, YLL, which accrues over remaining life expectancy at time of death, was discounted using a 5% discount rate based on assumptions of expected future economic growth in Africa.^34^ In a sensitivity analysis, we assessed the impact of undiscounted YLL and a higher discount rate on our results.

### Sensitivity analyses

Deterministic and probabilistic sensitivity analyses (DSA and PSA) were conducted to assess uncertainty in model inputs of our base case analysis (i.e. the pairwise comparison between each of the two COAVs and usual care). The DSA varied individual parameters sequentially over a specified range (Online Appendix 1 Table S2) whilst holding all other parameters constant at their base case value.

Probabilistic sensitivity analyses were conducted to assess the robustness of the results simultaneously, to uncertainty in model parameters. This was conducted by fitting appropriate distributions (Table 1) to each parameter^35^ and running 10 000 Monte Carlo simulations that drew parametric inputs from these distributions.^36^ For the PSA, standard errors of each parameter were used to determine the range but when not reported in data sources, standard errors were assumed to be 20% of the mean value. For the DSA, 95% confidence intervals estimated from the standard errors of each parameter were used as the range.

### Scenario analyses

Scenario analyses were conducted to assess the impact of structural changes in the model on the results of our base case analysis. Scenarios considered include (Table 2):

- Cost-effectiveness in COVID-19 vaccinated patients (Scenario 1): Given that COVID-19 vaccination programmes have been implemented in our study countries, we assessed the cost-effectiveness of COAVs in vaccinated patients. However, evidence from a randomised control trial (RCT) suggests that Molnupiravir does not reduce the risk of hospitalisation in vaccinated patients at high risk of disease progression.^37^ Whilst there is no reported RCT evidence on the efficacy of Paxlovid in vaccinated populations, an observational study suggests that Paxlovid effectively reduces the risk of hospitalisation in vaccinated individuals.^38^ Therefore, in this scenario, we assessed the cost-effectiveness of Paxlovid in vaccinated populations using effectiveness estimates from the observational study.^38^ Furthermore, although each country’s base case disease parameters are likely to reflect both high natural immunity and COVID-19 vaccine effectiveness, we adjusted base case hospitalisation rates downwards using vaccine efficacy^39^ and vaccination coverage of each study country.^14^ (Table 3).
- One hundred per cent probability of early treatment initiation (Scenario 2): In the base case analysis, we accounted for the likelihood of initiating treatment after 5 days of symptom onset, for which we assumed zero treatment efficacy. To assess the impact of treatment initiation on our results, we modelled a scenario that assumed a 100% probability of early treatment initiation in all three study countries.
- Inclusion of productivity losses (Scenario 3): A broader perspective was adopted to account for productivity losses in the acute phase of COVID-19 disease and premature death. Time lost from work was valued using each study country’s GDP per capita^40^ and adjusted for unemployment rate^41^ over a period equivalent to the duration of illness for surviving patients, and up to retirement age^42,43^ for patients who die (Table 3). Productivity losses due to COVID-19 death were discounted using a 5% discount rate.^34^
- Post-acute impact of COVID-19 (Scenario 4): Patients with severe COVID-19 are likely to be readmitted to hospital following the initial acute phase of the disease.^44^ We extended the model to account for readmission 1 year following the acute disease phase (Online Appendix 1 Figure S3). We assumed one hospital readmission during this time and obtained relevant data on readmission rates and length of hospitalisation from existing literature.^44,45^ The cost of readmission was assumed to be the average cost of inpatient care for COVID-19, whilst disability weight for post-acute disease phase was based on disability weights recommended by the European Burden of Disease Network Consensus COVID-19 models^31^ (Table 3).
- Inclusion of future unrelated health care costs (Scenario 5): To account for future unrelated health care costs, all surviving cases were allocated an annual cost over their remaining life expectancy valued at each country’s health expenditure per capita^40^. All future unrelated healthcare costs were discounted using a 5% discount rate.^34^

**TABLE 2:** Scenario description.

Scenario	Description
Base case	Target population – COVID-19 unvaccinated patients. Assumes country-specific test rate as a proxy for probability of treatment initiation within 5 days of symptom onset. Does not account for productivity losses due to COVID-19 illness and premature deaths. Does not account for readmissions in the first year following the acute disease phase. Does not account for future unrelated healthcare costs.
Scenario 1	Target population: COVID-19 vaccinated patients.
Scenario 2	Assumes 100% probability of treatment initiation within 5 days of symptom onset.
Scenario 3	Accounts for productivity losses due to COVID-19 illness and premature death.
Scenario 4	Accounts for readmissions in the first year following the acute disease phase.
Scenario 5	Accounts for future unrelated healthcare costs.

**TABLE 3:** Model parameters (scenario analyses).

Parameters	Ghana	Rwanda	Zambia	Data source
**Scenario 1**				
Vaccine effectiveness	0.972	0.972	0.972	Zheng et al.^39^
Vaccination coverage	0.35	0.68	0.12	Edouard et al.^14^
**Vaccine weighted hospitalisation rates (%)**				
All adult population	0.018	0.0045	0.0177	Authors estimation
Adults ≥ 65 years old	0.162	0.0407	0.159	Authors estimation
Adults with other risk factors	0.0541	0.0136	0.0530	Authors estimation
COAV effectiveness in vaccinated individuals (Paxlovid)				
Relative risk, hospitalisation	0.54	0.54	0.54	Najjar-Debbiny et al.^38^
**Scenario 3**				
**Productivity loss: due to acute illness ($)**				
Outpatient care only	28.80	8.36	12.65	The World Bank^40^, Trading Economics^41^, Nyabor^42^, Rwanda Ministry of Local Government^43^
Mild or moderate, survived	17.40	5.05	7.64	The World Bank^40^, Trading Economics^41^, Nyabor^42^, Rwanda Ministry of Local Government^43^
Mild or moderate, died	29.82	8.66	13.10	The World Bank^40^, Trading Economics^41^, Nyabor^42^, Rwanda Ministry of Local Government^43^
Severe, survived	29.58	8.59	12.99	The World Bank^40^, Trading Economics^41^, Nyabor^42^, Rwanda Ministry of Local Government^43^
Severe, died	17.57	5.10	7.72	The World Bank^40^, Trading Economics^41^, Nyabor^42^, Rwanda Ministry of Local Government^43^
Critical, survived	29.58	8.59	12.99	The World Bank^40^, Trading Economics^41^, Nyabor^42^, Rwanda Ministry of Local Government^43^
Critical, died	17.57	5.10	7.72	The World Bank^40^, Trading Economics^41^, Nyabor^42^, Rwanda Ministry of Local Government^43^
**Productivity loss: due to premature death ($)**				
All adult population	24 774.47	10 462.95	10 883.96	The World Bank^40^, Trading Economics^41^, Nyabor^42^, Rwanda Ministry of Local Government^43^
Adults ≥ 65 years old	0	0	0	The World Bank^40^, Trading Economics^41^, Nyabor^42^, Rwanda Ministry of Local Government^43^
Adults with comorbidities	24 774.47	10 462.95	10 883.96	The World Bank^40^, Trading Economics^41^, Nyabor^42^, Rwanda Ministry of Local Government^43^
GDP per capita ($)	2363.30	822.30	1137.30	The World Bank^40^
Unemployment rate	0.047	0.205	0.13	Trading Economics^41^
Compulsory retirement age (years)	60	65	60	(Nyabor^42^, Rwanda Ministry of Local Government^43^) Assumed for Zambia
**Scenario 4**				
Proportion readmitted	0.069	0.069	0.069	Ramzi^44^
Number of readmissions	1	1	1	Assumption
Readmission length of hospital stay (days)	12	12	12	Sotoodeh Ghorbani et al.^45^
Mortality risk following survival with long-term sequalae	0.04	0.04	0.04	Ramzi^44^
Disability weight: Post-acute of COVID-19	0.22	0.22	0.22	Wyper et al.^31^
Disability duration (years)	1	1	1	Assumption
Cost of readmissions ($)	1171.75	1066.11	1181.00	Ismaila et al.^28^, Torres-Rueda et al.^29^
**Scenario 5**				
**Future unrelated health care costs ($)**				
All adult population	3022.74	1969.91	2899.33	The World Bank^40^
Adults ≥ 65 years old	1492.38	970.22	1475.18	The World Bank^40^
Adults with comorbidities	3022.74	1969.91	2899.33	The World Bank^40^

Note: Please see the full reference list of the article Edoka IP, Drake T, Baker P, et al., A cost-effectiveness analysis of Molnupiravir and Paxlovid for outpatient treatment of COVID-19 in three African countries. J Public Health Africa. 2025;16(1), a805. https://doi.org/10.4102/jphia.v16i1.805, for more information.

COAV, COVID-19 oral antivirals; GDP, gross domestic product; $, United States dollar.

### Ethical considerations

This article followed all ethical standards for research without direct contact with human or animal subjects.

## Results

### Pairwise comparison with usual care

The results varied across the three countries and target populations (Table 4). In the all-adult patient target population, across all three countries, Paxlovid and Molnupiravir were both more costly and more effective compared to usual care, resulting in ICERs ranging from $2302.00 per DALY averted in Rwanda to $26 107.00 in Ghana for Paxlovid and from $12 000.00 per DALY averted in Rwanda to $78 166.00 in Ghana for Molnupiravir (Table 4, Panel A).

**TABLE 4:** Cost-effectiveness of Paxlovid and Molnupiravir in Ghana, Rwanda and Zambia.

Treatment options	GHANA				RWANDA				ZAMBIA			
	DALYs	Costs ($)	ICER ($)		DALYs	Costs ($)	ICER ($)		DALYs	Costs ($)	ICER ($)	
			Compared to usual care	Full incremental analysis			Compared to usual care	Full incremental analysis			Compared to usual care	Full incremental analysis
**A. All adults**												
Usual care	0.2981	178	-	-	0.227	108.0	-	-	0.429	149	-	-
Molnupiravir	0.2978	196	78 166	ED	0.226	121.0	12 000	SD	0.428	165	24 614	SD
Paxlovid	0.2973	198	26 107	26 107	0.224	116.0	2302	2302	0.427	164	6944	6944
**B. Elderly ≥ 65 years**												
Usual care	0.3720	821	-	SD	0.301	502.0	-	SD	0.498	688	-	SD
Molnupiravir	0.3700	824	1024	SD	0.291	462.0	D	SD	0.492	672	D	SD
Paxlovid	0.3650	801	D	D	0.269	374.0	D	D	0.480	621	D	D
**C. Adults with other risk factors**												
Usual care	0.3170	339	-	-	0.244	206.2	-	SD	0.451	284	-	SD
Molnupiravir	0.3160	353	20 307	SD	0.241	206.1	D	SD	0.449	292	4099	SD
Paxlovid	0.3140	349	4260	4260	0.233	180.1	D	D	0.444	278	D	D

Note: Cost-effectiveness threshold: Ghana = $433.25; Rwanda = $246.50; Zambia = $503.50.

D, dominant; SD, strictly dominated; ED, extendedly dominated; DALY, disability-adjusted life year; ICER, incremental cost-effectiveness ratios; $, United States dollar.

In the elderly population (Table 4, Panel B), Molnupiravir and Paxlovid were both less costly and more effective compared to usual care (i.e. Molnupiravir and Paxlovid dominated usual care) in Rwanda and Zambia. In Ghana, Paxlovid dominated usual care, whilst Molnupiravir was both more costly and more effective, resulting in an ICER of $1024.00 per DALY averted (Table 4, Panel B).

In adults with other risk factors (Table 4, Panel C), Paxlovid dominated usual care in Rwanda and Zambia and was both more costly and more effective than usual care in Ghana, resulting in an estimated ICER of $4260.00 per DALY averted (Table 4, Panel C); Molnupiravir dominated only in Rwanda, and in Ghana and Zambia, ICERs were estimated at $20 307.00 and $4099.00 per DALY averted, respectively (Table 4, Panel C).

**FIGURE 1:**
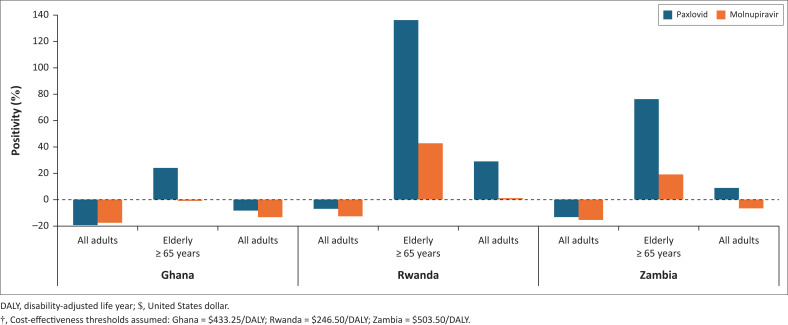
Incremental net monetary benefits?, Paxlovid and Molnupiravir compared to usual care.

Figure 1 presents INMBs of Paxlovid and Molnupiravir compared to usual care estimated at the cost-effectiveness threshold assumed for each country (Table 1). Except for the all-adult target population in Ghana, INMB of Paxlovid (vs usual care) was higher than INMB of Molnupiravir (vs usual care) across all target populations and study countries (Figure 1).

### Incremental cost-effectiveness analysis

The full incremental analysis (Table 4) ranked the three treatment options from highest to lowest DALYs lost. In the all-adult target population, Paxlovid dominated Molnupiravir across all three study countries, and ICERs for Paxlovid compared to usual care were estimated at $26 107.00 in Ghana, $2302.00 in Rwanda and $6944.00 in Zambia (Table 4). In the elderly patients, Paxlovid dominated both usual care and Molnupiravir in Ghana, Rwanda and Zambia. In adults with other risk factors, Paxlovid dominated Molnupiravir in Ghana, and an ICER of $4260.00 was estimated for Paxlovid vs. usual care, whilst in Rwanda and Zambia, Paxlovid dominated both usual care and Molnupiravir (Table 4).

### Sensitivity analysis

The results of the one-way deterministic sensitivity analyses, estimated using INMBs, are shown in Online Appendix 1 Figure S4. Across all study countries, three parameters had the largest impact on the base results for Paxlovid–COAV treatment costs, the likelihood of treatment initiation within 5 days of symptom onset, and hospitalisation rate modifiers affecting hospitalisation rates for the elderly population and adults with other risk factors (Online Appendix 1 Figure S4). For example, in Ghana, at a lower hospitalisation rate multiplier in the elderly population (1.8 vs. 9, Online Appendix 1 Table S2), the INMB for Paxlovid became negative at a cost-effectiveness threshold of $430.00 per DALY averted, whilst at a higher hospitalisation rate multiplier (16.2 vs. 9, Online Appendix 1 Table S2), the INMB for Paxlovid became positive in adults with other risk factors. At lower Paxlovid treatment cost ($5.00 vs $25.00, Online Appendix 1 Table S2), the INMB for Paxlovid in Ghana became positive in the all-adult target population and in adults with other high-risk factors for disease progression. In Rwanda and Zambia, Paxlovid treatment cost, hospitalisation rate modifiers and the likelihood of early treatment initiation similarly impact the base case INMBs.

Across the three study countries and target populations, uncertainty in model parameters followed a similar pattern for Molnupiravir where COAV treatment cost, likelihood of early treatment initiation, hospitalisation rate modifier and in addition, Molnupiravir treatment effect had the largest impact on the base case results for Molnupiravir (Online Appendix 1 Figure S4).

The results of the probabilistic sensitivity analysis are summarised in cost-effectiveness acceptability curves (Online Appendix 1 Figure S5–S7). Compared to other target populations, the proportion of cost-effective simulations was highest in the elderly population (Online Appendix 1 Figures S6). Conversely, in the all-adult population, the proportion of simulations that were cost-effective was very low across all study countries – approximately 0% for Molnupiravir in all study counties and 0%, < 15% and 0% for Paxlovid in Ghana, Rwanda and Zambia, respectively, at cost-effectiveness thresholds assumed for each country (Online Appendix 1 Figure S5). In patients with other risk factors, the PSA results were also mixed – across study countries, the proportion of simulations falling below the cost-effectiveness threshold assumed was 0%, < 50% and < 10% for Molnupiravir in Ghana, Rwanda and Zambia, respectively and < 10%, approximately 100% and < 80% for Paxlovid in Ghana, Rwanda and Zambia, respectively (Online Appendix 1 Figure S7).

### Scenario analysis

The results of the scenario analysis are displayed in Online Appendix 1 Table S4. Across all three countries and target populations modelled, the results for Paxlovid and Molnupiravir are largely unaffected by the inclusion of productivity losses, model extension to account for readmission and the inclusion of future unrelated health costs (Online Appendix 1 Table S4). However, the results differed substantially from the base case analysis for scenarios 1 (COVID-19 vaccine effectiveness) and 2 (100% probability of early treatment initiation). When COVID-19 vaccine effectiveness is accounted for in the model (scenario 1), the ICERs for Paxlovid for all study countries increased across all target populations, with the INMB remaining positive only for elderly patients in Rwanda and Zambia. When a 100% probability of early treatment is assumed in scenario 2, Paxlovid and Molnupiravir dominated usual care in all three study countries and in all target populations modelled except for Molnupiravir in the all-adult target population in Rwanda.

## Discussion

In this study, we assessed the cost-effectiveness of Paxlovid and Molnupiravir in three target populations as well as assessed scenarios and parameters that may affect the cost-effectiveness of COAV in the African setting.

Overall, our analysis suggests that Paxlovid is likely to be cost-effective for unvaccinated patients at high risk of severe outcomes in a range of African settings. The INMB for Paxlovid was observed to be consistently higher than the INMB for Molnupiravir suggesting that the additional benefits generated per cost is higher for Paxlovid. This was similarly observed in the full incremental analysis where Paxlovid dominated both Molnupiravir and usual care in elderly patients (across all study countries) and adults with other risk factors (in Rwanda and Zambia). However, the results of a direct comparison between Molnupiravir and Paxlovid in the full incremental analysis should be interpreted with caution given differences in the trial designs and baseline characteristics of trial participants.^5,6,13^ For example, participants with previous exposure to COVID-19 were excluded from the trial assessing the efficacy of Paxlovid but included in the trial for Molnupiravir.^5,6,13^ Furthermore, both trials were conducted at different time periods. Given the rapidly evolving nature of the pandemic at the time of these studies both standard of care and predominant COVID-19 variant were different in the two trial periods. As a result, a direct comparison of the efficacies of Molnupiravir and Paxlovid has a high degree of uncertainty, the characterisation of which in a probabilistic sensitivity analysis is unlikely to address the fundamental contextual difference between the two trials.^46^

The findings of the base case analysis were significantly affected by key model parameters that varied across the three study countries modelled. For example, in Ghana and Zambia where a lower likelihood of early treatment initiation (7% and 18%, respectively) was assumed, Molnupiravir was not cost-effective in patients with other high-risk factors but dominated usual care in Rwanda where a higher early treatment initiation likelihood (41%) was assumed. The results were also sensitive to the modelled baseline hospitalisation rates. In elderly patients for whom we assumed the highest risk of disease progression, both Paxlovid and Molnupiravir were cost-saving across all study countries regardless of likelihood of early treatment initiation, except for Ghana, where Molnupiravir was not cost-effective at the cost-effective threshold assumed. Neither Paxlovid nor Molnupiravir were cost-effective in the unvaccinated all-adult target population across all study countries. This is likely because of the lower risk of hospitalisation modelled in this target population. However, when a 100% likelihood of early treatment initiation was assumed, Molnupiravir and Paxlovid were cost-saving in the unvaccinated all-adult target population for all three study countries except Rwanda, which had the lowest baseline hospitalisation rate modelled. However, the finding in the all-adult population should be interpreted with caution given that clinical efficacy estimates for both Molnupiravir and Paxlovid apply only to populations at high risk of disease progression.^5,6^

In vaccinated patients, who are at a reduced risk of severe disease, hospitalisation and death,^47^ our finding suggests that Paxlovid could be cost-effective only in elderly patients particularly in settings with a higher likelihood of treatment initiation within 5 days of symptom onset as seen in Rwanda and Zambia. However, Paxlovid is not likely to represent good value for money in the other study target populations because of the reduced effectiveness of Paxlovid in these populations^38^ and the lower hospitalisation rates modelled. These findings are based on effectiveness estimates drawn from an observational study,^38^ which may be subject to a higher degree of bias because of non-randomisation of patients. Therefore, these findings should be reassessed as new evidence from RCTs emerges.

Overall, our findings are based on the assumption that Paxlovid and Molnupiravir could be accessed at lower prices. However, as highlighted in the DSA, COAV treatment costs could impact on the findings reported here such that at treatment costs similar to high-income countries, Paxlovid (at approximately $600.00 per treatment course) and Molnupiravir (at approximately $700.00 per treatment course) will not be cost-effective for any target population in our study settings.

From the probabilistic sensitivity analysis, our base case findings are robust to joint uncertainty in model parameters more so for Paxlovid vs. usual care which had the highest probability of being cost-effective across all study countries.

Notwithstanding contextual differences between settings, our findings are consistent with those from other settings^9,10,11,46^ where Paxlovid and Molnupiravir compared to usual care in pairwise analyses, are cost-effective in high-risk populations with mild or moderate COVID-19. Despite comparatively lower hospitalisation rates seen in Africa, Paxlovid could be cost-effective in limited use cases, if offered at lower prices for unvaccinated sub-populations at high risk of disease progression.

This study has some limitations. Due to a dearth of context-specific data, the study relied on proxies and plausible assumptions for key model parameters – hospitalisation rates were largely drawn from studies in the first year of the pandemic. However, in the current era of the milder variants and high natural immunity, COVID-19 hospitalisation rates have substantially decreased across all settings. Although we accounted for this by adjusting hospitalisation rates downward, we cannot rule out that our base case hospitalisation rates remain too high. In the absence of context-specific costs of the clinical management of COVID-19 in Rwanda and Zambia, we relied on costs estimates from a large multi-country extrapolation study.^29^ Although we adjusted these cost estimates to be more comparable to clinical management costs from single-country studies, these estimates may not be the true representation of the disease management cost in Rwanda and Zambia. Nevertheless, the results of the one-way sensitivity analysis suggest that our results were robust to uncertainty in clinical management costs for Rwanda and Zambia. Unlike previous cost-effectiveness analyses of COAVs, this study explicitly modelled early treatment initiation on the cost-effectiveness of COAVs using total COVID-19 test performed per 1000 population as a proxy for the likelihood of early treatment initiation. Although testing rates are useful indicators of the stringency of COVID-19 control measures within countries, without knowing the timing of testing, it is unclear if test rates suitably capture the probability of early diagnosis and treatment initiation. A lower (higher) probability of early treatment initiation is likely to reduce (increase) the cost-effectiveness of the COAV. However, except for model results for Molnupiravir in the elderly and other risk groups in Ghana and Rwanda, respectively, the results of the one-way sensitivity analysis were largely robust to uncertainty in our proxy for early treatment initiation.

## Conclusion

Paxlovid has the potential to be a cost-effective treatment option in African countries similar to Ghana, Rwanda or Zambia for the limited use case of unvaccinated patients at high risk of severe disease progression. More evidence is needed to determine cost-effectiveness for unvaccinated patients who have previously been infected with COVID-19 and may have developed some immune protection. Whilst this evidence is based on three countries, the diversity of key parameters across the three study countries allowed for explicit modelling of nuances relevant to the African setting. Therefore, this study provides useful insights to policymakers on a range of context-specific factors that should be considered and adapted when making funding decisions on COAVs based on findings from cost-effectiveness analyses.
